# The fate of lignin during hydrothermal pretreatment

**DOI:** 10.1186/1754-6834-6-110

**Published:** 2013-08-01

**Authors:** Heather L Trajano, Nancy L Engle, Marcus Foston, Arthur J Ragauskas, Timothy J Tschaplinski, Charles E Wyman

**Affiliations:** 1 Department of Chemical and Environmental Engineering and Center for Environmental Research and Technology, Bourns College of Engineering, University of California Riverside, 1084 Columbia Ave, Riverside, CA 92507, USA; 2 Current address: Department of Chemical and Biological Engineering, The University of British Columbia, 2360 East Mall, Vancouver, British Columbia V6T 1Z3, Canada; 3 Biosciences Division, Oak Ridge National Laboratory, PO Box 2008 MS6341, Oak Ridge, TN 37831, USA; 4 School of Chemistry and Biochemistry, Institute of Paper Science and Technology, Georgia Institute of Technology, 500 10th Street N.W., Atlanta, GA 30332, USA; 5 Department of Energy, Environmental & Chemical Engineering, Washington University in St. Louis, 1 Brookings Drive, Saint Louis, MO 63130, USA; 6 BioEnergy Science Center, Oak Ridge National Laboratory, PO Box 2008 MS6341, Oak Ridge, TN 37831, USA

**Keywords:** Condensation, Depolymerization, Flowthrough pretreatment, Hydrothermal pretreatment, Lignin-carbohydrate complex, Phase transition

## Abstract

**Background:**

Effective enzymatic hydrolysis of lignocellulosic biomass benefits from lignin removal, relocation, and/or modification during hydrothermal pretreatment. Phase transition, depolymerization/repolymerization, and solubility effects may all influence these lignin changes. To better understand how lignin is altered, *Populus trichocarpa* x *P. deltoides* wood samples and cellulolytic enzyme lignin (CEL) isolated from *P. trichocarpa x P. deltoides* were subjected to batch and flowthrough pretreatments. The residual solids and liquid hydrolysate were characterized by gel permeation chromatography, heteronuclear single quantum coherence NMR, compositional analysis, and gas chromatography–mass spectrometry.

**Results:**

Changes in the structure of the solids recovered after the pretreatment of CEL and the production of aromatic monomers point strongly to depolymerization and condensation being primary mechanisms for lignin extraction and redeposition. The differences in lignin removal and phenolic compound production from native *P. trichocarpa x P. deltoides* and CEL suggested that lignin-carbohydrate interactions increased lignin extraction and the extractability of syringyl groups relative to guaiacyl groups.

**Conclusions:**

These insights into delignification during hydrothermal pretreatment point to desirable pretreatment strategies and plant modifications. Because depolymerization followed by repolymerization appears to be the dominant mode of lignin modification, limiting the residence time of depolymerized lignin moieties in the bulk liquid phase should reduce lignin content in pretreated biomass. In addition, the increase in lignin removal in the presence of polysaccharides suggests that increasing lignin-carbohydrate cross-links in biomass would increase delignification during pretreatment.

## Background

The development of transportation fuels with low greenhouse gas emissions is imperative due to growing demand for transportation fuels, decreasing conventional petroleum supplies, and increasing evidence of global climate change. Ethanol produced by fermentation of sugars contained in cellulose and hemicellulose in cellulosic biomass could address all of these challenges, but its production requires expensive pretreatments and enzymes. Furthermore, efficient enzymatic hydrolysis requires hydrothermal or other pretreatments that alter the composition and/or structure of biomass [1]. One of the primary plant cell wall components is lignin, an amorphous, phenolic polymer which strengthens the cell wall and protects the plant from microbial damage [2,3]. Because delignification improves enzymatic hydrolysis of the remaining biomass [4], lignin removal is an oft-cited goal for pretreatment, but many cost-effective pretreatments do not lower lignin content appreciably. However, they do modify lignin to make biomass more accessible to enzymes, and a better understanding of the mechanisms of lignin alterations during pretreatment can provide valuable insights into new pretreatment or plant modification strategies.

**Figure 1:**
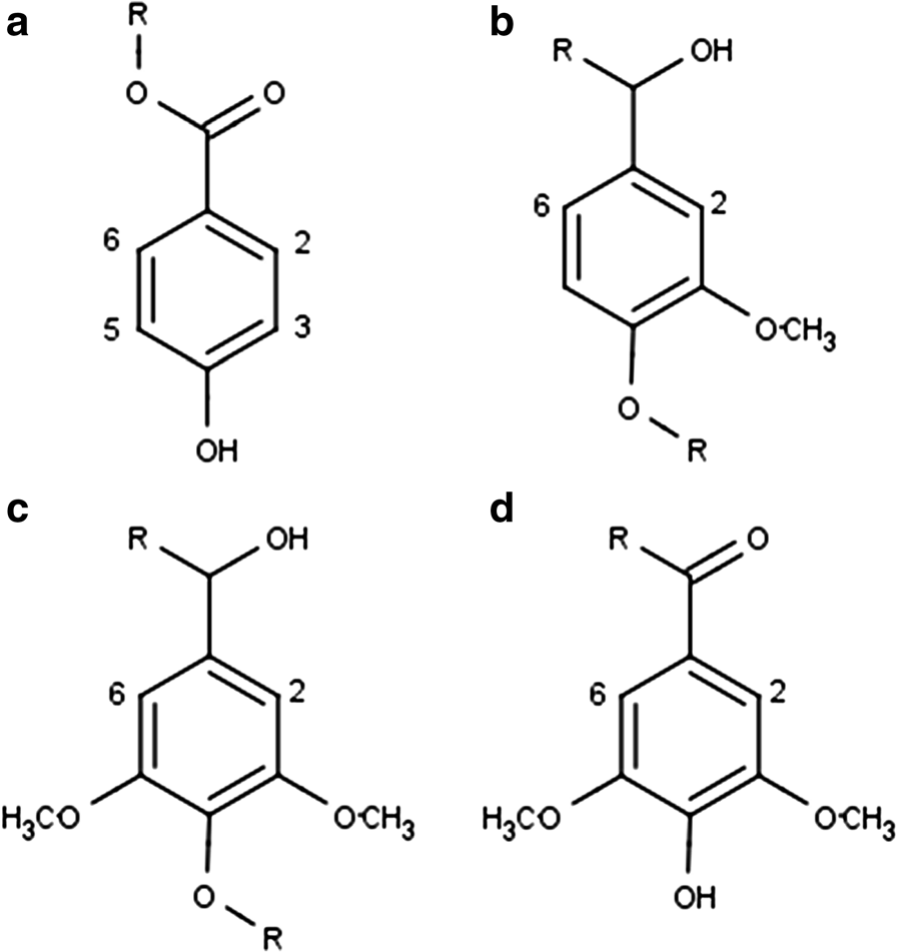
Molecular structures of selected hardwood lignin monomers: (a) p-hydroxybenzoate, (b) guaiacyl, (c) syringyl, and (d) oxidized syringyl units.

**Figure 2:**
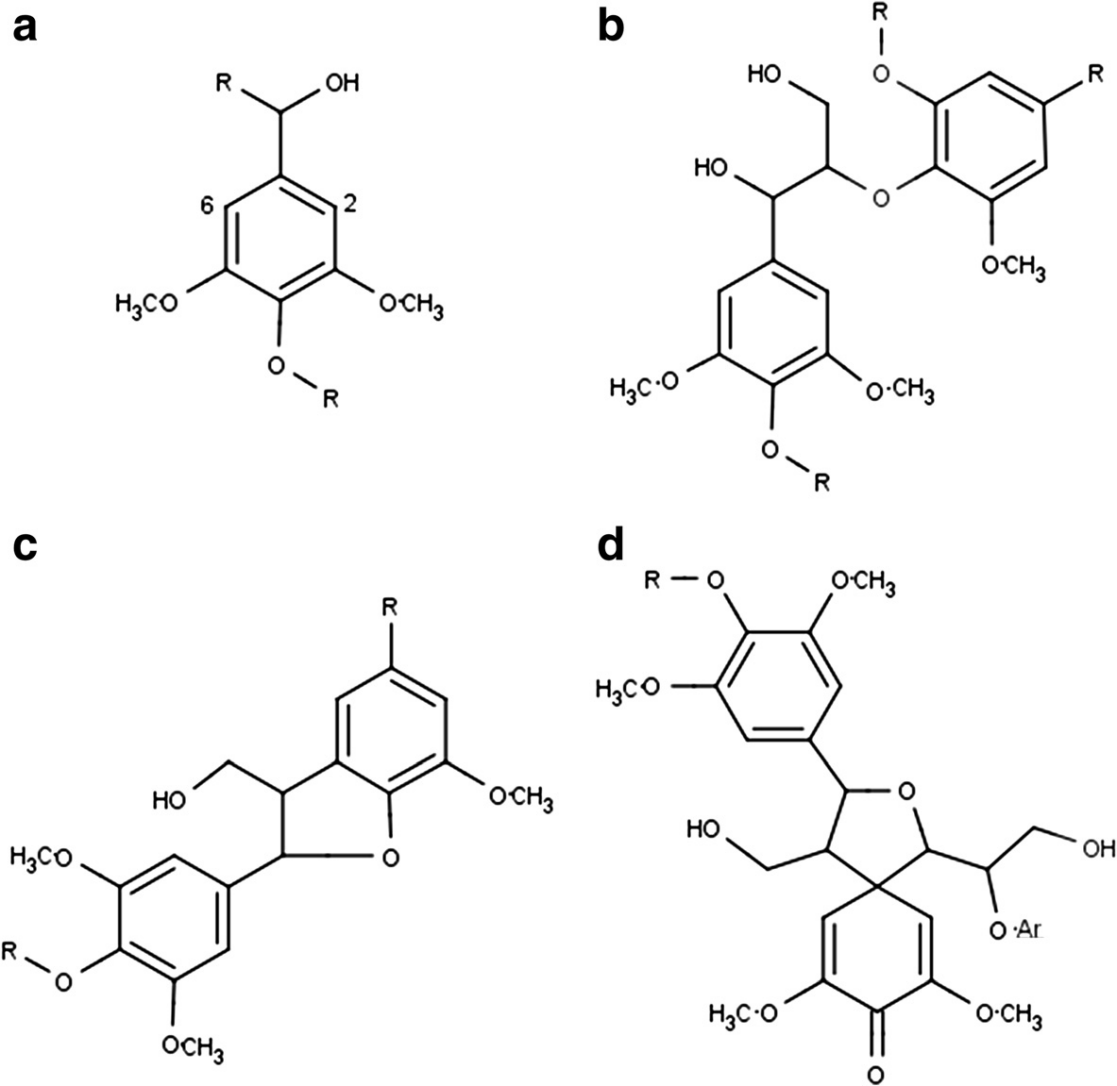
**Selected hardwood lignin structures: (a) methoxy group, (b) β-O-4 ether, (c) *β*-5/*α*-*O*-4 phenyl-coumaran, and (d) spirodienone.**

The primary monomeric structural units of lignin are *p*-coumaryl, coniferyl, and sinapyl alcohol (Figure 1) [3,5]. Hardwoods, such as *Populus,* typically contain syringyl and guaiacyl lignin synthesized from sinapyl and coniferyl alcohol, respectively [5], and β-O-4 (β aryl ether) linkages account for approximately 80% of the linkages involving syringyl units [6]. Other linkages, such as β-5/α-*O*-4 phenyl-coumaran and spirodienone linkages, are also present, as shown in Figure 2. As the cell wall is lignified, ester, ether, and glycosidic bonds form between lignin and polysaccharides, resulting in lignin-carbohydrate complexes (LCC) [3,6]. Noncovalent interactions may also link lignin and hemicellulose, but there are few interactions between lignin and cellulose in native biomass [6].

Lignin cycles between the solid and liquid phase during pretreatment through a complex mechanism that may involve phase transition, reaction, and/or solubilization. Many researchers [7-11] have observed droplets, determined to be lignin [7], on a wide variety of biomass types, including corn stover, switchgrass, wheat straw, and *Tamarix ramosissima,* following hydrothermal or dilute acid pretreatment and hypothesized that these droplets form as a result of the transition of lignin from glassy state to rubbery state, followed by coalescence, migration, and extrusion from the cell wall [7]. Upon cooling, these droplets harden. This view is somewhat incomplete since the effects of increasing the temperature of an amorphous solid are complex. When an amorphous solid is heated, it passes through a glass transition stage over a range of temperatures [12,13]. An amorphous solid without cross-linking will undergo rubbery flow in the absence of thermal degradation, while a cross-linked polymer, such as lignin, can only undergo rubbery flow after bonds break [12]. A review of the literature indicates the glass transition of lignin occurs somewhere in the range between 80 and 193°C [12,14-19]; the breadth of this range reflects differences in biomass, sample moisture content, lignin isolation procedures, and analytical techniques [12,16].

In addition to these morphological changes, lignin reacts during pretreatment. Under acidic conditions, carbonium ion intermediates are formed with a high affinity for nucleophiles within the lignin structure [3]. Hydrolysis leads to depolymerization, while reactions between the carbonium ions and nucleophiles leads to repolymerization or condensation [20,21]. Evidence of depolymerization during pretreatment includes the loss of β-O-4 bonds [21,22] and a decrease in the molecular weight of lignin at extended pretreatment times [23,24]. Extensive cleavage of β-O-4 bonds without high yields of lignin monomers suggests depolymerization is accompanied by repolymerization [25]. Additional evidence of repolymerization includes an increase in molecular weight during short pretreatments [21,23,24], an increase in lignin carbon-carbon bonds, as shown by infrared spectroscopy [24], and alkaline nitrobenzene oxidation [26]. There are few kinetic models of lignin depolymerization. However, as lignin is a solid phase reactant, the rate of depolymerization is likely proportional to the area of the solid–liquid interface [27]:

(1) $$\;r=k''\rho_{p}A_{\mathrm{surf}}f\left(C\right)\;$$

where *k''* is the rate constant per unit surface area, *ρ*_*p*_ is the particle density, *A*_*surf*_ is the surface area, and *f(C)* is some function of reactant concentration.

Evidence also suggests that the presence of carbohydrates influences the solubility of lignin during pretreatment. The addition of carbohydrates, such as pectin or arabinoxylan during *in vitro* synthesis of artificial lignin or dehydrogenation polymer (DHP), increased the molecular weight of the resulting DHP [28,29], likely through the formation of hydrophobic complexes between DHP and carbohydrates, which prevented precipitate growth [28-30]. Similar hydrophobic aggregates or the covalent bonds between lignin and hemicellulose may improve lignin solubility during lignin deconstruction as well. When corn stover was subjected to flowthrough pretreatment, there was a linear relationship between xylan and lignin removal, leading to the hypothesis that lignin is released to solution as part of an LCC, and once in solution, the bonds within the LCC break, producing lignin and carbohydrate fragments [31-33].

Observing these changes as a function of time is challenging in traditional batch reactors. It is particularly difficult to follow product evolution as a function of time since side and degradation reactions generate products such as those known as humins that interfere with lignin characterization [34,35]. Additionally, quenching batch reactors to stop a reaction may create artifacts such as precipitation of oligomers [36]. These problems are avoided with a fixed bed flowthrough reactor. In this system, because solubilized products are quickly and continuously removed from the reactor products can be tracked as a function of time, the potential for side and degradation reactions is limited, and few solubilized products are present in the reactor as the reaction is quenched.

Cellulolytic enzyme lignin (CEL) isolated from *Populus trichocarpa x P. deltoides* was pretreated in batch and flowthrough systems in order to study the changes in molecular weight, chemical bonds, and functional groups of lignin during pretreatment as well as the production of soluble aromatic compounds. The differences in lignin removal and soluble aromatic products from *P. trichocarpa x P. deltoides* wood pretreated at the same conditions were examined to determine the impact of lignin-carbohydrate bonds on lignin deconstruction. These insights into the fundamental phenomena may suggest new plant modifications and pretreatment strategies.

## Results and discussion

### Changes in the molecular weight of cellulolytic enzyme lignin following hydrothermal pretreatment

The relative number and weight average molecular weights of CEL were determined before and after pretreatment using gel permeation chromatography and calculated from Equations (2) and (3):

(2) $${\overset{—}{M}}_{n}=\frac{\sum_{i}N_{i}M_{i}}{\sum_{i}N_{i}}$$

(3) $${\overset{—}{M}}_{w}=\frac{\sum_{i}N_{i}M_{i}^{2}}{\sum_{i}N_{i}M_{i}}$$

in which N_i_ is the number of polymers detected having molecular weight M_i_.

**Figure 3:**
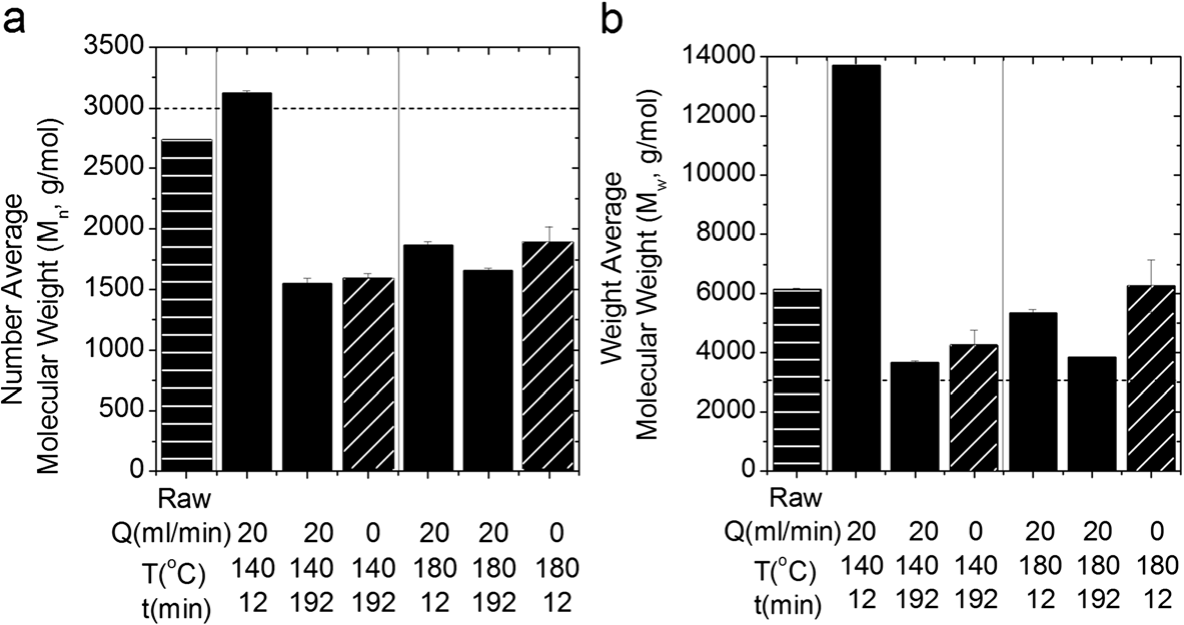
**Molecular weights of cellulolytic enzyme lignin (CEL) as determined by gel permeation chromatography: (a) number average and (b) weight average.** Molecular weights of CEL before pretreatment, after flowthrough pretreatment, and after batch pretreatment are shown with horizontal hatching, solid, and diagonal hatching, respectively. The black dashed line represents a molecular weight of 3000 on both panels.

The results in Figure 3 for batch (0 mL/min) and flowthrough (20 mL/min) pretreatments show that the number average molecular weight of the CEL pretreated at the same temperature and time but under flow and batch conditions were similar, while the weight average molecular weights of the batch pretreated CEL were 16 to 18% higher. Since the weight average molecular weight is more sensitive to the presence of large polymers, these results indicate more long chain polymers in the batch pretreated CEL than flow pretreated CEL. Solids had equal residence times during batch and flowthrough pretreatment, however soluble compounds had a very short residence time, 0.15 min, during flowthrough pretreatment. Thus, while solid phase reactions, such as depolymerization, could proceed continuously during both pretreatment types, liquid phase reactions, such as repolymerization, were limited during flowthrough pretreatment. The increase in the solids’ molecular weight after flowthrough pretreatment at 140°C for 12 minutes also suggested repolymerization reactions. Others [21,23,24] have reported increases in the molecular weight of lignin following batch pretreatment.

### Changes in the structure and composition of cellulolytic enzyme lignin following hydrothermal pretreatment

**Figure 4:**
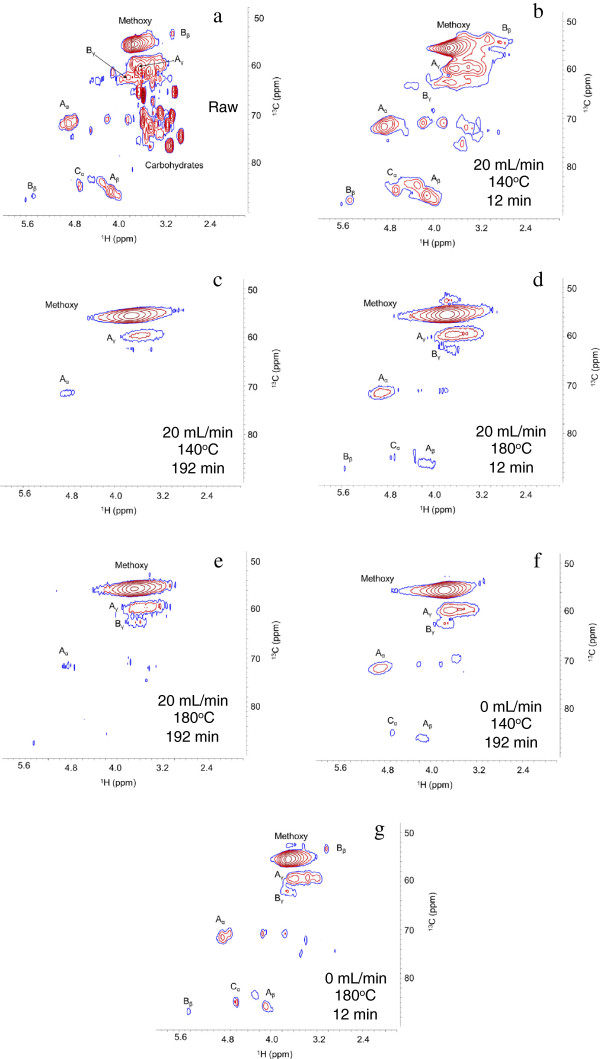
**HSQC NMR spectra of raw and pretreated cellulolytic enzyme lignin in the aliphatic region: (a) raw; (b) 20 mL/min, 140°C, 12 min; (c) 20 mL/min, 140°C, 192 min; (d) 20 mL/min, 180°C, 12 min; (e) 20 mL/min, 180°C, 192 min; (f) 0 mL/min, 140°C, 192 min; (g) 0 mL/min, 180°C, 12 min.** Identified units include methoxy groups, β-*O*-4 ethers (A_α_, A_β_, A_γ_), β-5/α-*O*-4 phenyl-coumararan (B_α_, B_β_, B_γ_), and spirodienone (C_α_) units. Signal assignments are presented in Table 4.

**Figure 5:**
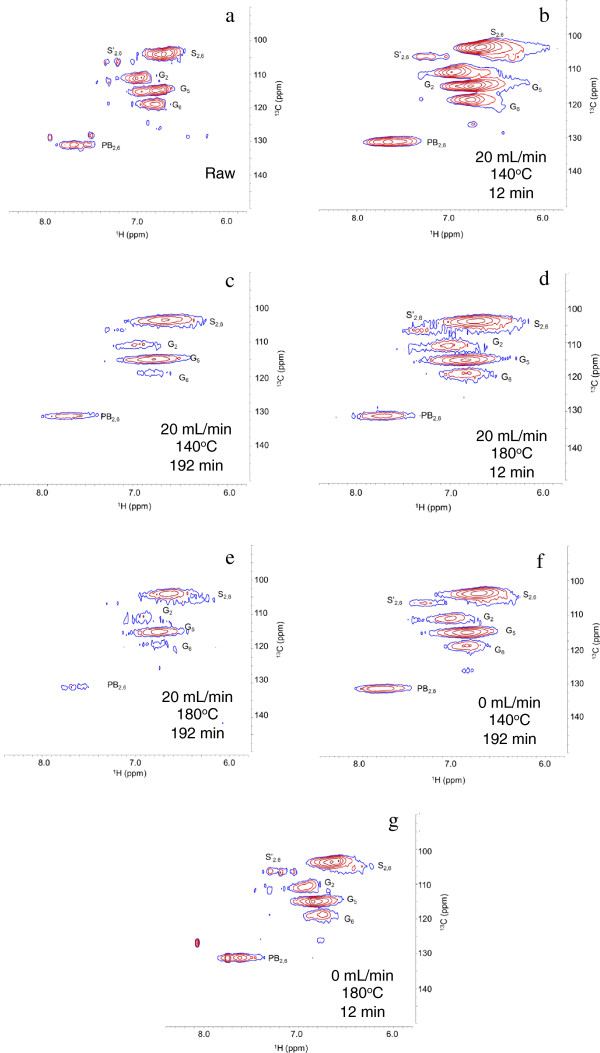
**HSQC NMR spectra of raw and pretreated cellulolytic enzyme lignin in the aromatic region: (a) raw; (b) 20 mL/min, 140°C, 12 min; (c) 20 mL/min, 140°C, 192 min; (d) 20 mL/min, 180°C, 12 min; (e) 20 mL/min, 180°C, 192 min; (f) 0 mL/min, 140°C, 192 min; (g) 0 mL/min, 180°C, 12 min.** Identified units include *p*-hydroxyphenyl (PB_2, 6_), guaiacyl (G_2_, G_5,_ G_6_), syringyl (S_2, 6_), and oxidized syringyl (S’_2, 6_) units. Signal assignments are presented in Table 4.

Heteronuclear single quantum coherence nuclear magnetic resonance (HSQC-NMR) and well-established lignin spectral assignments [37] were applied to examine the inter-unit linkages and aromatic and aliphatic chemical moieties in CEL. HSQC-NMR was not applied to the *Populus* samples because the carbohydrate signatures would obscure the lignin-related signals. Although methods for semi-quantification have been proposed [38,39], HSQC-NMR is inherently difficult to quantitate for a polymer system for a variety of reasons, as outlined by Zhang et al [40]. Moreover, because of the difficulties in dissolving CEL for NMR, the results in Figures 4 and 5, representing DMSO soluble fraction, are discussed qualitatively.

The spectra in Figure 4 reveal that residual carbohydrates detected in the spectra of the raw CEL were not present in the spectra of the pretreated materials, indicating that the residual carbohydrates were removed or reacted to produce pseudo-lignin [35]. However, because no Klason lignin was detected in holocellulose, the cellulose-hemicellulose fraction of the *P. trichocarpa x P. deltoides* samples, pretreated at the same conditions, pseudo-lignin formation during these pretreatments is unlikely. In both Figures 4 and 5, cross-peaks in the spectra (recorded under identical conditions) of the pretreated materials were broad and distorted compared to untreated CEL due to a low signal to noise ratio and altered nuclear relaxation (as indicated by significant line-broadening [40]). This outcome was attributed to altered solubility and a modified lignin structure with low local molecular mobility, presumably due to condensation reactions. Finally, the relative intensity of signals resulting from the pretreated spectra were lower in comparison to signals in the spectra of the raw CEL. In Figure 4, the reduction in signal intensity indicates the loss of methoxy groups, β-O-4 ether bonds, β-5/α-O-4 phenyl-coumaran bonds, and spirodienone bonds, while the changes shown in Figure 5 indicate loss of functional groups. These results are similar to those seen by Samuel et al. [37,38] and Yelle et al. [41].

**Figure 6:**
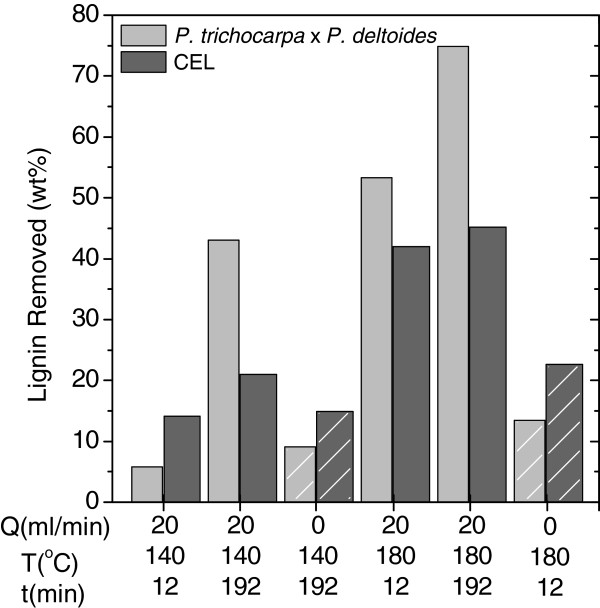
**Percent lignin removal from *Populus trichocarpa x P. deltoides *wood samples and cellulolytic enzyme lignin (CEL) after batch (0 mL/min, diagonal hatching) and flowthrough (20 mL/min, solid) pretreatments at 140 and 180°C for 12 and 192 minutes.** Percent lignin removal was calculated from the Klason lignin and total solids mass balance. See Additional file 1, Additional file 2, Additional file 3, and Additional file 4 for details of the calculations.

**Figure 7:**
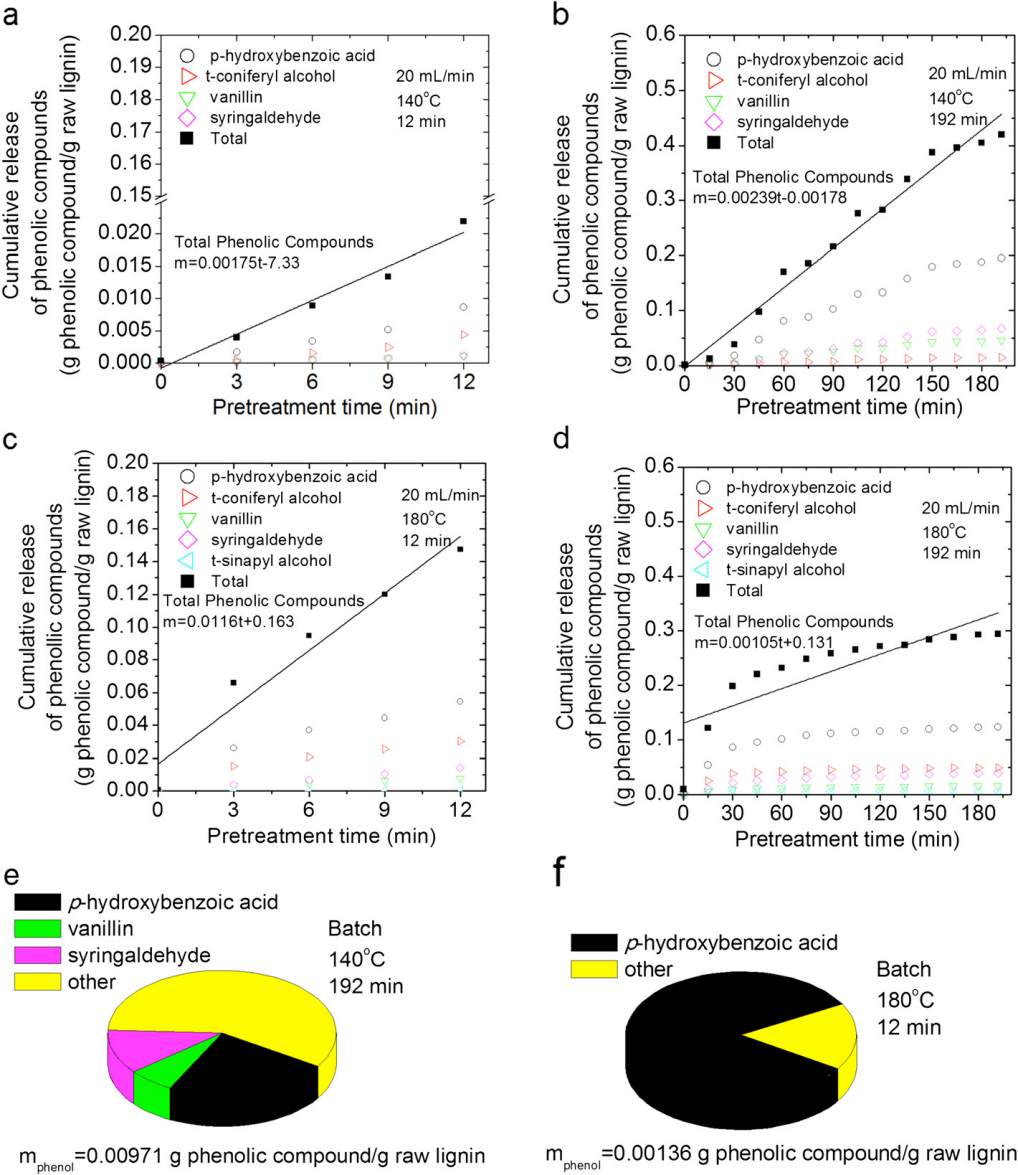
**Phenolic compounds in hydrolysate generated by flowthrough and batch pretreatment of cellulolytic enzyme lignin.** Cumulative release of phenolic compounds from flowthrough pretreatment (20 mL/min) are plotted as a function of time: **(a)** 140°C, 12 minutes; **(b)** 140°C, 192 minutes; **(c)** 180°C, 12 minutes; **(d)** 180°C, 192 minutes. Distribution of primary phenolic compounds after batch pretreatment: **(e)** at 140°C for 192 min and **(f)** at 180°C for 12 min.

**Figure 8:**
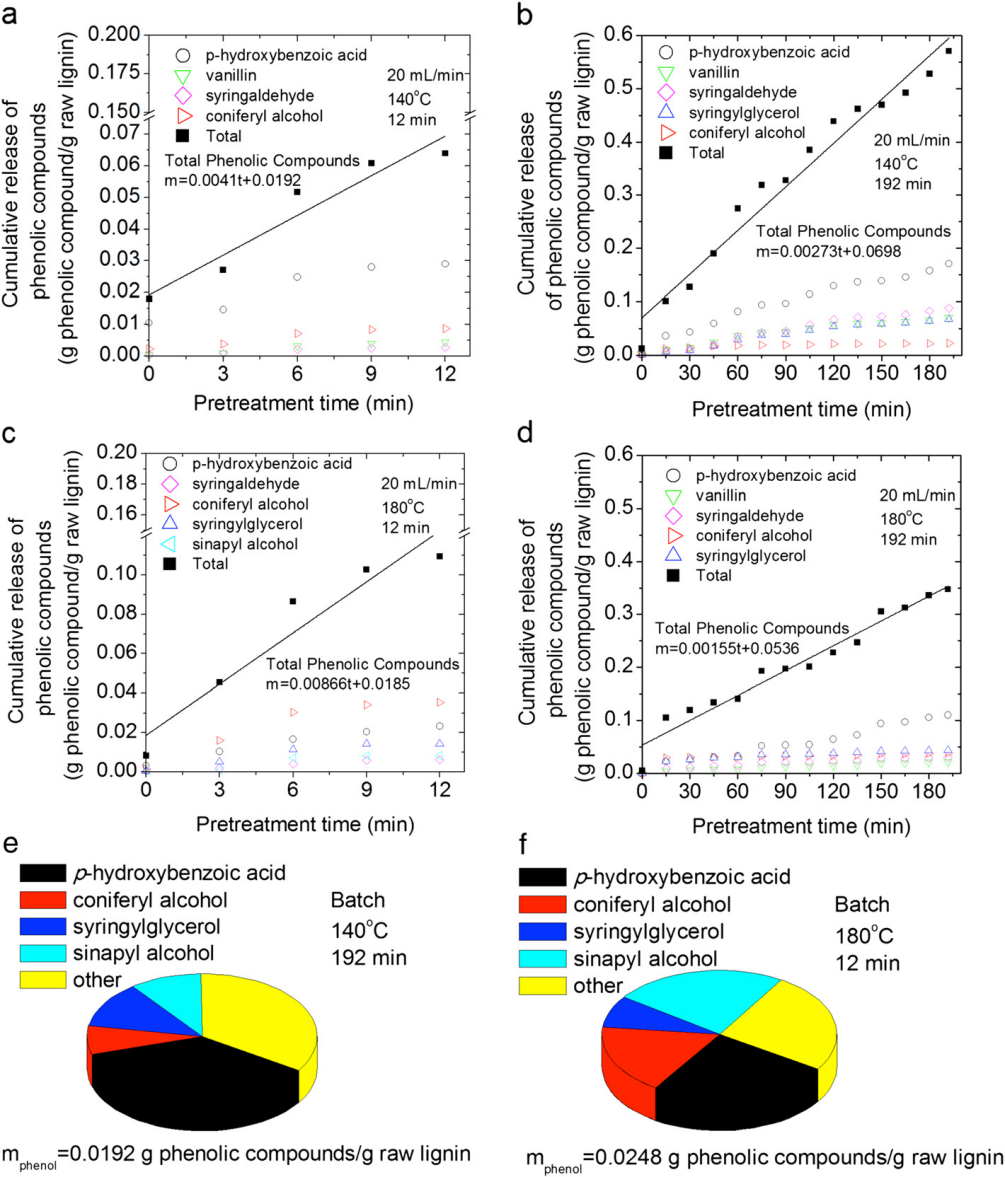
**Phenolic compounds in hydrolysate generated by flowthrough and batch pretreatment of *Populus trichocarpa x P. deltoides *wood samples.** Cumulative release of phenolic compounds from flowthrough pretreatment (20 mL/min) are plotted as a function of time: **(a)** 140°C, 12 min; **(b)** 140°C, 192 min; **(c)** 180°C, 12 min; **(d)** 180°C, 192 min. Distribution of primary phenolic compounds after batch pretreatment: **(e)** at 140°C for 192 min and **(f)** at 180°C for12 min.

The relatively more flexible aliphatic functionality found in significant proportions in the untreated CEL is generally associated with more prevalent monolignol inter-unit linkages, such as β-O-4 ether bonds; therefore its loss is typically correlated with degradation or depolymerization. However, the loss of this type of inter-unit linkage combined with the significant mass loss data (Figure 6) and production of phenolic compounds (Figures 7 and 8) suggests that the observed molecular weights (Figure 3) for the pretreated lignin were higher than one might expect. This was particularly evident for CEL pretreated at 140°C for 12 minutes for which the loss of mass and aliphatic functional groups was accompanied by an increase in molecular weight. Changes in solubility and alterations in nuclear relaxation, the latter indicating reduction in local molecular mobility, confirm the presence of large polymers, which, when combined with loss of ether bonds and aliphatic functionality, suggest that repolymerization is also occurring. Lignin repolymerization of this nature results in a more condensed polymer structure, primarily composed of carbon-carbon bonds, in agreement with results from previous studies [24,26].

### Lignin removal during hydrothermal pretreatment of cellulolytic enzyme lignin and *Populus trichocarpa x P. deltoides*

Lignin removal from CEL and *P. trichocarpa x P. deltoides* wood samples by pretreatment was determined from the difference in the mass of lignin initially loaded into the reactor and the mass of lignin recovered following pretreatment (Figure 6, see Additional file 1, Additional file 2, Additional file 3 and Additional file 4 for calculation details). More lignin was removed from both substrates during flowthrough pretreatment than during batch pretreatment at the same temperature and time. This indicates the flow of water removes lignin fragments from the reactor before they are redeposited, confirming that flowthrough pretreatment facilitates the observation of product formation as a function of time. Lignin removal from CEL by flowthrough pretreatment at 140°C for 12 minutes was approximately equal to lignin removal from CEL by batch pretreatment at 140°C for 192 minutes, which also suggests that interrupting the redeposition of lignin to the solid phase can shorten pretreatment times without compromising lignin removal.

Figure 6 shows that batch pretreatment removed more lignin from CEL than from *P. trichocarpa x P. deltoides*, possibly due to the formation of pseudo-lignin from carbohydrates [35]. However, no Klason lignin was detected in holocellulose pretreated at the same conditions therefore pseudo-lignin formation is unlikely. The difference may instead be due to the difficulty in recovering pretreated CEL from the reactor. More lignin was removed from *P. trichocarpa x P. deltoides* samples than from CEL by three of four flowthrough pretreaments (Figure 6: 140°C/192 min, 180°C/12 min, 180°C/192 min). *P. trichocarpa x P. deltoides* samples remained as discrete particles during pretreatment but pretreated CEL formed a solid which molded to the shape of the reactor therefore, differences in reactive surface area could lead to differences in delignification, in line with Equation (1). Alternatively, the presence of polysaccharides could cause increased lignin removal from *P. trichocarpa x P. deltoides* samples. To examine surface area effects, all parameters in Equation (1) were assumed constant except the surface area. Furthermore, based on the appearance of the residual solids, the pretreatment of *P. trichocarpa x P. deltoides* was modeled as a bed of nonporous spherical lignin particles with constant diameter, and no adjustments were made for the presence of polysaccharides. The CEL bed during flowthrough pretreatment was modeled as a tube with an outer radius *r*_*rxtr*_ and an inner radius of *0.5r*_*rxtr*_. The solid interface during batch pretreatment of CEL was modeled as a solid cylinder with only the upper surface available for reaction. The ratios of these surface areas and the ratios of percent lignin removed by pretreatment at 180°C are presented in Table 1. Comparison of the surface area ratios and the percent lignin removed ratios reveals that differences in surface areas were far too great relative to the differences in mass removal to account for the differences in lignin removal from *P. trichocarpa x P. deltoides* and CEL samples. Therefore, similar to the addition of carbohydrates during lignin synthesis [28,29], the presence of carbohydrates increased the extent of lignin extraction during flowthrough pretreatment. This is also in agreement with previous work by Foston et al. [31] and Liu and Wyman [32,33]. However, the mildest flowthrough pretreatment at 140°C for 12 min removed more lignin from CEL than from *P. trichocarpa x P. deltoides*. The heterogeneity of biomass results in a distibution of lignin-carbohydrate and lignin-lignin bond types and strengths. At these mild conditions, the weakest lignin-lignin bonds in the CEL may break while lignin-carbohydrate bonds in *P. trichocarpa x. P. deltoides* remain intact, thus limiting lignin release. As pretreatment time increases, the proposed solubility effect of carbohydrates becomes significant.

**Table 1:** **Ratio of surface area or percent lignin removed during pretreatment of *Populus trichocarpa x P. deltoides *or cellulolytic enzyme lignin**

**Ratio**	**Surface area**	**Lignin removed**
	**(*A***_***surf, i***_***/A***_***surf, j***_**)**	**(*m***_***i***_***/m***_***j***_**)**
**TD, F/CEL, B**	1209	2.36
**TD, F/ CEL, F**	384	1.27
**CEL, F/ CEL, B**	3	1.86

The surface areas of solids and percent lignin removed during flowthrough pretreatment of *Populus trichocarpa x P. deltoides* wood samples (*i, j* = TD, F) and cellulolytic enzyme lignin in flowthrough (*i, j* = CEL, F) and batch pretreatment (*i, j* = CEL, B) at 180°C for 12 min were calculated and compared.

### Production of phenolic compounds from the hydrothermal pretreatment of cellulolytic enzyme lignin and *Populus trichocarpa x P. deltoides*

Gas chromatography mass-spectrometry (GCMS) was used to detect phenolic compounds in the hydrolysate. The cumulative release of total phenolic compounds normalized to the mass of raw lignin is plotted as a function of flowthrough pretreatment time for each run with CEL (Figure 7) and *P. trichocarpa x P. deltoides* (Figure 8). The cumulative release of key individual phenolic compounds is also presented. The distribution of phenolic compounds produced by batch pretreatment of CEL (Figure 7) and *P. trichocarpa x P. deltoides* (Figure 8) are also shown as pie charts along with the total normalized mass of phenolic compounds detected. In total, eighteen phenolic monomers, including hydroquinone, *p*-hydroxybenzoic acid, coniferyl alcohol, *p*-coumaryl alcohol, 5-hydroxyconiferyl alcohol, vanillic acid, protocatechuic acid, syringic acid, syringylglycerol (erythro and threo), coniferyl aldehyde, sinapyl aldehyde, syringaresinol, vanillin, syringaldehyde, sinapyl alcohol, medioresinol, and pinoresinol were identified. Eighteen additional compounds detected at low concentrations are likely also phenolic compounds, but definitive identifications could not be made.

There were more monomers in the hydrolysates from flowthrough pretreatment than batch pretreatment, regardless of substrate (Figures 7, 8). As with the observations of solids removal, this result confirms that flowing water interrupts lignin repolymerization. When this observation is considered in tandem with other results from batch pretreatment of CEL: low solids removal, high weight average molecular weight, and loss of characteristic bonds and side chains, the case for the condensation reactions becomes very strong. The CEL product profile after batch pretreatment at 140°C was much more diverse than after batch pretreatment at 180°C; in fact, *p*-hydroxybenzoic acid accounted for 83% of the phenolic compounds produced during batch pretreatment at 180°C. This result suggests that the condensation of phenolic compounds, with the exception of *p*-hydroxybenzoic acid, was much greater at 180°C during batch pretreatment. Greater repolymerization at 180°C was also supported by the fact that the weight average molecular weight of CEL recovered from batch pretreatment at 180°C was slightly higher than the weight average molecular weight of CEL recovered from batch pretreatment at 140°C (Figure 3b).

Comparison of the production rate and total mass of phenolic compounds produced during flowthrough pretreatment of both substrates at 140°C and 180°C for 12 minutes revealed, unsurprisingly, that phenolic compounds were produced more rapidly and in greater amounts at 180°C. However, it is surprising that the production rate and total mass of phenolic compounds released after 192 minutes of flowthrough pretreatment at 140°C was greater than the rate and mass of phenolic compounds produced at 180°C. Since the total mass of lignin removed during flowthrough pretreatment at 180°C was greater than the total mass removed by flowthrough pretreatment at 140°C (Figure 6) and the molecular weights of the CEL solids recovered from these runs were similar (Figure 3), it is unlikely that condensation reactions were accelerated relative to depolymerization reactions at 180°C. Instead, the lower mass of phenolic compounds at 180°C could be the result of the higher temperature allowing for the production of larger, soluble phenolic oligomers that were undetectable by GCMS (>1000 Da) due to their low volatility.

*p*-Hydroxybenzoic acid was the primary phenol observed in the hydrolysate for the majority of runs. Without HSQC analysis of *P. trichocarpa x P. deltoides* samples, the relative content of *p*-hydroxybenzoate units to syringyl and guaiacyl units is unknown. However, the HSQC spectra of raw CEL in Figure 5 show that CEL contains fewer *p*-hydroxybenzoate groups than syringyl and guaiacyl units. Regardless, based on the CEL results, *p*-hydroxybenzoate groups were released more easily than syringyl or guaiacyl groups.

It has been previously suggested that due to greater propensity for covalent linkages, guaiacyl units are less easily extracted than syringyl units during hydrothermal pretreatment [26,37,42]. It has also been shown that due to the lack of a methoxy group in the C5 position, guaiacyl units undergo condensation reactions more easily [26]. This hypothesis is supported by greater coniferyl alcohol production during flowthrough pretreatment at 180°C relative to pretreatment at 140°C. Also in agreement with the hypothesis, the *P. trichocarpa x P. deltoides* hydrolysates contain more syringaldehyde, syringylglycerol, syringylglycerol glycoside, and sinapyl alcohol than coniferyl alcohol and vanillin, but contradictorily, the CEL hydrolysates contain more coniferyl alcohol and vanillin relative to sinapyl alcohol and syringaldehyde. Therefore, the differences in the relative amounts of guaiacyl and syringyl type products from CEL and *P. trichocarpa x P. deltoides* samples are strong evidence that cross-linking between lignin and hemicellulose changes the relative reactivity of guaiacyl and syringyl units.

Lora and Wayman [24] found that autohydrolysis of *Populus tremuloides* proceeded more slowly than autohydrolysis of milled wood lignin and attributed this difference to the reaction of carbohydrate degradation products with lignin products. However in this study, with one exception, more phenolic compounds were produced from *P. trichocarpa x P. deltoides* samples than from CEL during identical pretreatments. Differences between the results of this study and those by Lora and Wayman [24] could be due to a number of factors. The lignin that Lora and Wayman used was produced by ball milling biomass for 18 days, followed by a dioxane water extraction, while the CEL used in this study was produced under less severe conditions (7 days ball milling, enzymatic digestion of carbohydrates), thus reducing the possibility of producing an isolated lignin that is more reactive than native lignin. In addition, Lora and Wayman’s work was conducted in a batch reactor for which condensation reactions are more significant. As previously noted in this paper, the very short space time of the flowthrough reactor limits production of degradation products and thus condensation reactions. Thus, assuming that CEL is more representative of native lignin, the greater production of phenolic compounds from *P. trichocarpa x P. deltoides* samples was likely due the presence of carbohydrates increasing lignin reactivity or solubility, as was shown during the *in situ* synthesis of lignin [28-30].

## Conclusions

Previous studies showed that during hydrothermal pretreatment, lignin cycles between the solid and liquid phase and suggested that depolymerization/repolymerization reactions and lignin-carbohydrate interactions play key roles, although the mechanisms were not well understood. In order to investigate lignin behavior during pretreatment, *Populus trichocarpa x P. deltoides* wood samples and cellulolytic enzyme lignin (CEL) isolated from *P. trichocarpa x P. deltoides* were subjected to batch and flowthrough hydrothermal pretreatment. The residual solids and liquid hydrolysate were characterized by a wide range of analytical techniques.

Pretreatment of CEL resulted in loss of characteristic lignin bonds and functional groups from the solids, the production of phenolic monomers, and modest reductions in molecular weight. These observations point strongly to depolymerization and condensation being the primary mechanisms for lignin extraction and redeposition. No direct evidence of phase transition was observed.

There were some similarities in the pretreatment of *P. trichocarpa x P. deltoides* wood samples and CEL. Short liquid residence times, due to the flow of water, limited condensation reactions for both substrates, as evidenced by greater lignin extraction by flowthrough pretreatment.

More lignin was extracted and more phenolic compounds were produced by pretreatment of *P. trichocarpa x P. deltoides* wood samples than by pretreatment of CEL. The difference in lignin extraction between the two substrates was too small to be due to differences in solid–liquid interfacial area. Therefore, it is likely that lignin-carbohydrate interactions significantly influence lignin deconstruction during pretreatment. Due to their greater potential for cross-linking, guaiacyl units are thought to be less easily extracted and more easily condensed, but guaiacyl based phenolic compounds were the dominant type produced from CEL. However, syringyl based phenolic compounds were the dominant aromatic constituents released from *P. trichocarpa x P. deltoides* wood samples*,* suggesting that cross-links between hemicellulose and lignin modify the reactivity of guaiacyl and syringyl groups.

The production of ethanol from lignocellulosic biomass requires efficient and economical pretreatment and enzymatic hydrolysis. Effective lignin removal or alteration during pretreatment leads to improved enzymatic hydrolysis. The knowledge of lignin depolymerization/repolymerization kinetics and the impact of lignin composition and lignin-carbohydrate interactions on lignin removal gained in this study will help guide plant modification and pretreatment strategies. An increase in lignin-carbohydrate cross-links in biomass and limiting the residence time of depolymerized lignin moieties during pretreatment would increase lignin removal during pretreatment.

## Methods

### Substrates

The stem wood of hybrid cottonwood poplar *Populus trichocarpa x P. deltoides* clone (TD) ‘53-239’ ♂ used in this study was provided by Oak Ridge National Laboratory, TN. Logs were debarked, split with an axe, chipped (Yard Machines 10HP, MTD Products Inc., Cleveland, OH), and knife milled (Model 4 Wiley Mill, Thomas Scientific, Swedesboro, NJ) through a 1 mm screen size; all of these operations were performed at the National Renewable Energy Laboratory (NREL). After one month of air-drying at NREL, the chips had a moisture content of approximately 5 wt%. The material was further milled to particles with dimensions of 0.18 mm to 0.85 mm (Thomas-Wiley Laboratory Mill Model 4, Arthur H. Thomas Company, Philadelphia, PA) before being shipped. The cellulolytic enzyme lignin (CEL) was isolated from *P. trichocarpa x P. deltoides* by modifying the procedures described by Chang et al. [43] and Björkman [44]. *P. trichocarpa x P. deltoides* was ball-milled for 7 days; this material was then subjected to two consecutive rounds of enzymatic hydrolysis with 500 IU cellulase/g *P. trichocarpa x P. deltoides* (Novozym 188, Sigma-Aldrich, St. Louis, MO) and 200 IU β-glucosidase/g *P. trichocarpa x P. deltoides* (Celluclast 1.5 L, Sigma-Aldrich, St. Louis, MO) for seventy-two hours at 50°C shaken at a frequency of 150 rpm. Following hydrolysis, the solids were washed and then subjected to two rounds of extraction with dioxane for twenty-four hours. The composition of the untreated substrates determined using the procedures described below are summarized in Table 2.

**Table 2:** **Composition of raw *Populus trichocarpa x P. deltoides *and cellulolytic enzyme lignin (CEL)**

**Component**	***P. trichocarpa x***	**Cellulolytic Enzyme Lignin (CEL)**
	*** P. deltoides***	
Glucan (wt%)	40.5 (0.2)	0.591 (0.034)
Xylan (wt%)	11.5 (0.4)	2.67 (0.08)
Klason lignin (wt%)	22.7 (0.1)	84.4 (2.3)

Values represent the average of triplicate samples. The standard deviations are shown in brackets.

### Reactors

**Figure 9:**
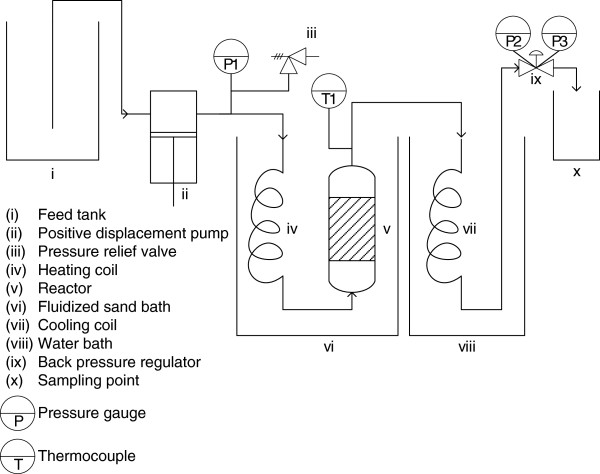
Schematic of the flowthrough pretreatment system.

A schematic of the flowthrough reactor system employed in this study is shown in Figure 9. A 2 L feed tank was used to hold the process water. A positive displacement pump (Prep100, LabAlliance, State College, PA) delivered water to the reactor, and the pressure of the system was set using the backpressure regulator (GO, Spartanburg, SC). The system pressure was monitored by pressure gauges P1 (US Gauge, max P 20.6 MPag), P2 (Ashcroft, max P 4.2 MPag), and P3 (Ashcroft, max P 4.2 MPag). The heating coil and reactor were heated using a fluidized sand bath (SBL-2D, Techne, Princeton, NJ), and the heating coil was a 2.6 m length of stainless steel tubing (D = 3.18 mm) with a coil diameter of 50.9 mm. The reactor was constructed of a stainless steel tube (D = 12.7 mm) with Swagelok fittings (SS-8-VCR-1, SS-8-VCR-3-8TA, SS-8VCR-6-810, SS-200-R-8, Swagelok, San Diego, CA). The total reactor length was 152 mm. The biomass was held in the reactor by 5 micrometre gaskets (SS-8-VCR-2-5 M, Swagelok, San Diego, CA). The system temperature was monitored with K-type thermocouple T1 at the reactor outlet and recorded as a function of time using a Digi-Sense DualLogR Thermocouple Meter (15-176-96, Fisher Scientific, Pittsburgh, PA). Data was transferred from the meter to a computer using an infrared adapter (EW-91100-85, Cole Parmer, Vernon Hills, IL). The cooling coil, a 5.3 m length of stainless steel tubing (D = 3.18 mm) with a coil diameter of 44.5 mm, was submerged in a 19 L water bath to cool the hydrolysate prior to sampling. The sampling point is open to the atmosphere so that hydrolysate could be collected continuously during a run.

Custom-built 10 mL reactors were used for the batch pretreatments. The reactors were constructed from stainless steel tubing with an outer diameter of 12.7 mm, a length of 150 mm, and sealed using threaded caps (SS-810-C, Swagelok, San Diego, CA). One reactor had a thermocouple (.062-K-U-4″-T3-10 ft TF/TF-MP, Wilcon Industries, Lake Elsinore, CA) inserted along the centerline to record the reactor temperature as a function of time using a Digi-Sense DualLogR Thermocouple Meter (15-176-96, Fisher Scientific, Pittsburgh, PA). Data was transferred from the meter to a computer using an infrared adapter (EW-91100-85, Cole Parmer**,** Vernon Hills, IL).

### Pretreatment

The pretreatment conditions, equipment set points, and sampling intervals for flowthrough and batch pretreatment are summarized in Table 3. These pretreatment conditions were selected in order to observe lignin deconstruction over a wide range of pretreatment severities. The flowthrough reactor was loaded with 0.71 g of dry CEL or 1.00 g of dry *P. trichocarpa x P. deltoides*. A representative flowthrough pretreatment run with CEL at 140°C (*T*_*rxtr*_) for 12 minutes is described; the same procedure was followed for all other runs as summarized in Table 3. The pump was primed, and the sand bath was heated to 149°C (*T*_*sand*_) prior to pretreatment. After the reactor was loaded with CEL it was attached to the flowthrough piping system. The pump was set to 20 mL/min (*Q*) and started, and the back pressure gauge was adjusted to 0.28 MPag (*P*_*set*_). The pressurized system was inspected for leaks at room temperature. After repairing any leaks, the reactor and heating coil were lowered into the sand bath, and the cooling coil was lowered into the water bath. A Digi-Sense DualLogR Thermocouple Meter was used to monitor the reactor temperature during the run. The time at which the temperature reached 138°C (*T*_*rxtr*_-2°C) was recorded as the start of the reaction. Hydrolysate was collected as the reactor was heated and then over 3 min (*Δt*) intervals during the run using pre-massed flasks. After each sampling interval, the filled flask was exchanged with a clean, pre-massed flask. The mass of the filled flask was recorded before a sample of the hydrolysate was taken for subsequent analysis. After 12 min, the reactor and heating coil were transferred to the water bath and cooled to 70°C, at which point the pump was stopped and the reactor removed from the piping. The residual solids in the reactor were collected by filtration, washed with three 100 mL volumes of deionized water, and dried at 45°C for 24 h. As the CEL solids had fused to the reactor, vigorous scraping was required to recover them.

**Table 3:** Summary of flowthrough (20 mL/min) and batch (0 mL/min) pretreatment conditions

**Flow rate**	**Temperature,**		**Set pressure**	**Time**	**Sample interval**
**( *Q *, mL/min)**	**Sand Bath (*T***_***sand***_**,°C)**	**Reactor (*T***_***rxtr***_**,°C)**	**(*P***_***set***_**, MPag)**	**(*t***_***rxn***_**, min)**	**( *Δt *, min)**
20	149	140	0.28	12	3
20	149	140	0.28	192	15
0	*T*_*sand, 1*_ *=* 142	140	n/a	192	n/a
	*T*_*sand, 2*_ *=* 140				
20	194	180	1.10	12	3
20	194	180	1.10	192	15
0	*T*_*sand, 1*_ *=* 182	180	n/a	12	n/a
	*T*_*sand, 2*_ *=* 180				

Batch pretreatment of CEL was conducted using one batch reactor loaded with 0.71 g CEL and 7.6 mL of deionized water while batch pretreatment of *P. trichocarpa x P. deltoides* samples was conducted using two tube reactors, each loaded with 0.44 g dry biomass and 7.88 mL of deionized water. A representative batch pretreatment of CEL at 140°C (*T*_*rxtr*_) is described; this procedure was repeated according to the conditions described in Table 3. The sand bath was heated to 142°C (*T*_*sand, 1*_). A batch reactor loaded with CEL and deionized water was sealed, shaken, and allowed to soak for 3.5 hours. A separate reactor equipped with a thermocouple was loaded with 8.34 mL deionized water. Both the tube reactor and thermocouple reactor were placed into a wire basket before being lowered into the sand bath, and the temperature was monitored with a Digi-Sense DualLogR Thermocouple Meter. The time at which the reactors reached 138°C (*T*_*rxtr*_-2°C) was taken as the start of the reaction, and the sand bath temperature was reset to 140°C (*T*_*sand, 2*_). After 192 minutes, the wire basket and reactors were transferred to a water bath and cooled to 70°C, and the temperature data was transferred to a computer. Following pretreatment, the residual solids were recovered by filtration using a pre-weighed crucible, and the filtrate was retained for analysis. Once again, vigorous scraping was required to remove CEL solids that had fused to the reactor walls. The pretreated solids were washed and dried as described above and were stored at room temperature until analyses were performed.

The following *P. trichocarpa x P. deltoides* pretreatments were done in triplicate in order to establish reproducibility: flowthrough pretreatment at 140°C for 192 min, flowthrough pretreatment at 180°C for 10 min, batch pretreatment at 140°C for 192 min, and batch pretreatment at 180°C for 12 min. The maximum standard deviations associated with the glucan, xylan, and lignin measurements during these runs were 0.6%, 3.2%, and 5.0%, respectively.

### Gel permeation chromatography

The relative number average and weight average molecular weights of CEL before and after pretreatment were determined by gel permeation chromatography (GPC), using the procedure described by Samuel et al. [37] Prior to GPC, the material was acetylated by combining 20 mg of lignin with approximately 1.0 mL of 1:1 anhydrous pyridine/acetic anhydride under an inert atmosphere for 48 h at room temperature. Adding 5 mg of ethanol quenched the reaction. The solvent was removed by rotary evaporation followed by drying in a vacuum oven at 45°C. Data were collected using an Agilent GPC SECurity 1200 system with a refractive index detector and a UV detector (270 nm) using tetrahydrofuran as an eluent at a flow rate of 1.0 mL/min. Four Waters Styragel columns (HR1, HR2, HR4, HR6) were used to separate acetylated lignin by molecular weight (Waters Co., Milford, MA). The calibration curve was constructed using eight polystyrene standards ranging in molecular weight from 1.5 × 10^3^ to 3.6 × 10^6^ g/mol (Polysciences Inc., Warrington, PA). The data was collected and processed using Polymer Standards Service WinGPC Unity software (Build 6807).

### Heteronuclear single quantum coherence nuclear magnetic resonance

Heteronuclear single quantum coherence nuclear magnetic resonance (HSQC NMR) was used to analyze the functional groups and intra-lignin bonds of CEL. Cross peaks were assigned according to Samuel et al. [37], as listed in Table 4. CEL was prepared for HSQC by dissolving 60 mg solids in 1 mL of dimethyl sulfoxide-d_6_ (DMSO-d_6_ 99.9 atom% D, Cambridge Isotope Laboratories, Andover, MA). However, given that complete dissolution of the pretreated CEL was not achieved, the results can only be interpreted qualitatively. 2D ^13^C-^1^H HSQC correlation NMR spectra were recorded on a Bruker DRX 500 spectrometer with a 5 mm z-gradient triple resonance probe with inverse geometry at 60°C (Bruker, Billerica, MA). Analysis was performed with a Bruker phase-sensitive gradient-edited HSQC pulse sequence using 1024 data points for a 0.11 s acquisition time, a 1.5 s recycle delay, a ^1^*J*_C-H_ coupling constant of 145 Hz, and acquisition of 256 data points in the F1 dimension. The data was processed using zero-filling to 2048 points and a typical squared sine-bell apodization in both F2 and F1 dimensions.

**Table 4:** **Assignment of **^**13**^**C-**^**1**^**H correlation signals detected in HSQC spectra of cellulolytic enzyme lignin isolated from *Populus trichocarpa x P. deltoides *wood samples**[37]

**δ**_**C**_**/δ**_**H **_**(ppm)**	**Assignment**
53.2/3.5	C_β_/H_β_ in phenylcoumarin substructure (B_β_)
53.6/3.1	C_β_/H_β_ in resinol (β-β) substructure
55.7/3.8	C/H in methoxyl group (Methoxy)
60.2/3.6	C_γ_/H_γ_ in β-O-4 substructure (A_γ_)
62.8/3.8	C_β_/H_β_ in phenylcoumarin substructure (B_β_)
71.5/4.8	C_α_/H_α_ in β-O-4 linkage (A_α_)
84.8/4.3	C_β_/H_β_ in β-O-4 linkage (A_β_)
81.4/5.1	C_β_/H_β_ in spirodienone substructure (C_β_)
84.7/4.7	C_α_/H_α_ in spirodienone substructure (C_α_)
87.1/5.5	C_α_/H_α_ in phenylcoumarin substructure (B_α_)
104.3/6.7	C_2,6_/H_2,6_ in etherified syringyl units (S_2,6_)
105.5/7.3	C_2,6_/H_2,6_ in oxidized C_α_=O (S'_2,6_)
113.7/6.3	C_β_/H_β_ in cinnamate unit (E_β_)
111.4/7.0	C_2_/H_2_ in guaiacyl units (G_2_)
115.4/6.77	C_5_/H_5_ in guaiacyl units (G_5_)
119.3/6.82	C_6_/H_6_ in guaiacyl units (G_6_)
130.0/7.5	C_2,6_/H_2,6_ in *p*-hydroxybenzoate units (PB_2,6_)
144.7/7.5	C_α_/H_α_ in cinnamate unit (E_α_)

### Carbohydrate and Klason lignin analysis

The structural carbohydrate and Klason lignin content of the raw *P. trichocarpa x P. deltoides* wood samples and CEL were determined using the two-step acid hydrolysis procedure outlined by Sluiter et al. [45] The composition of the pretreated *P. trichocarpa x P. deltoides* was also measured. However, since the CEL fused during pretreatment to form a single solid particle, the composition of pretreated CEL could not be measured. The carbohydrate composition of the hydrolysate produced by pretreatment was determined according the procedure outlined by Sluiter et al. [46] Sugars were detected by high pressure liquid chromatography (HPLC) using an Aminex HPX-87H column (BioRad, Hercules, CA) heated to 65°C with a separation module (Alliance 2695, Waters, Milford, MA) equipped with a refractive index detector (2414, Waters, Milford, MA). The eluent was 0.005 M sulfuric acid in the isocratic mode. These results were used to complete mass balances of glucan, xylan, and Klason lignin for each substrate-pretreatment combination (see Additional file 1, Additional file 2, Additional file 3 and Additional file 4).

### Gas chromatography–mass spectrometry

The phenolic compounds in the hydrolysates from pretreatment of CEL and *P. trichocarpa x P. deltoides* wood samples were determined by gas chromatography–mass spectrometry (GCMS) [47,48]. In brief, the samples were first filtered through a 0.45 μm nylon membrane and then dried in a helium stream. Sorbitol (15 μl of a 1 mg/mL aqueous solution) was added as an internal standard. The dried extracts were silylated to produce trimethylsilyl derivatives prior to injection on an Agilent Technologies Inc. 5975C inert XL gas chromatograph-mass spectrometer. Key mass/charge (m/z) ratios for identified aromatic metabolites were extracted from the total ion current to quantify metabolites free from co-eluting interference. Predetermined scaling factors were used to scale the extracted peak areas back up to the total ion current. The concentrations were normalized to the quantity of the internal standard (sorbitol) recovered, amount of sample derivitized, and injected. Predetermined response factors for each identified aromatic metabolite relative to the internal standard were used to determine actual metabolite concentration (μg/mL).

## Abbreviations

Asurf: Surface area; B: Batch pretreatment; CEL: Cellulolytic enzyme lignin; DHP: Dehydrogenation polymer; DMSO: Dimethyl sulfoxide; f(C): Function of reactant concentration; F: Flowthrough pretreatment; GCMS: Gas chromatography mass-spectrometry; GPC: Gel permeation chromatography; HSQC NMR: Heteronuclear single quantum coherence nuclear magnetic resonance; k'': Rate constant per unit surface area; LCC: Lignin-carbohydrate complexes; Mi: Molecular weight of polymer *i*; ${\bar{M}}_{n}$: Number average molecular weight; ${\bar{M}}_{w}$: Weight average molecular weight; Ni: Number of polymers with molecular weight *M*_*i*_; Q: Flow rate; R: Rate of reaction; rrxtr: Reactor radius; rxn: Reaction; t: Time; T: Temperature; TD: *Populus trichocarpa x P. deltoides*; ρp: Particle density.

## Competing interests

CEW is founding Editor in Chief of this Journal. CEW was cofounder of Mascoma Corporation and until recently, Chief Development Officer and Chair of their Scientific Advisory Board. CEW is also member of the Scientific Advisory Board of Mendel Biotechnology, Inc.

## Authors’ contributions

HLT performed the pretreatments and structural carbohydrate and Klason lignin analyses, and drafted the manuscript. NLE and TJT performed the gas chromatography–mass spectrometry. MF performed the gel permeation chromatography and heteronuclear single quantum coherence nuclear magnetic resonance. AJR and CEW coordinated the research and helped finalize the manuscript. All authors read and approved the final manuscript.

## Supplementary Material

Additional file 1 **Mass balances for the pretreatment of cellulolytic enzyme lignin at 140°C.** Additional file 1 summarizes the glucan, xylan, and Klason lignin mass balances for the pretreatment of cellulolytic enzyme lignin (CEL) at 140°C. The CEL fused during pretreatment to form a single solid particle therefore it was not possible to perform a compositional analysis on the residual solids. The concentrations of sugars in the hydrolysate were too low to accurately measure. However, as no carbohydrate signals were present in the HSQC-NMR spectra of the pretreated CEL (Figure 4) and no lignin was detected in holocellulose, the cellulose-hemicellulose fraction of the *P. trichocarpa x P. deltoides* samples, pretreated at the same conditions, it was assumed that the glucan and xylan were completely removed during pretreatment. Therefore the initial mass of glucan and xylan was substracted from the change in solid mass to determine the mass of lignin removed. Click here for file

Additional file 2 **Mass balances for the pretreatment of cellulolytic enzyme lignin at 180°C.** Additional file 2 summarizes the glucan, xylan, and Klason lignin mass balances for the pretreatment of cellulolytic enzyme lignin (CEL) at 180°C. The mass balances were calculated using the same process described for Additional file 1. Click here for file

Additional file 3 **Mass balances for the pretreatment of *Populus trichocarpa x P. deltoides *at 140°C.** Additional file 3 summarizes the glucan, xylan, and Klason lignin mass balances for the pretreatment of *Populus trichocarpa x P. deltoides* at 140°C. The mass of lignin removed was calculated as the difference between the Klason lignin in the untreated and pretreated solids. Click here for file

Additional file 4 **Mass balances for the pretreatment of *Populus trichocarpa x P. deltoides *at 180°C.** Additional file 4 summarizes the glucan, xylan, and Klason lignin mass balances for the pretreatment of *Populus trichocarpa x P. deltoides* at 180°C. The mass of lignin removed was calculated as the difference between the Klason lignin in the untreated and pretreated solids. Click here for file
