# Supplementary figures and images for: The fate of lignin during hydrothermal pretreatment
Source: Biotechnol Biofuels. 2013 Aug 1;6:110. doi: 10.1186/1754-6834-6-110 (PMC3751430; doi:10.1186/1754-6834-6-110)

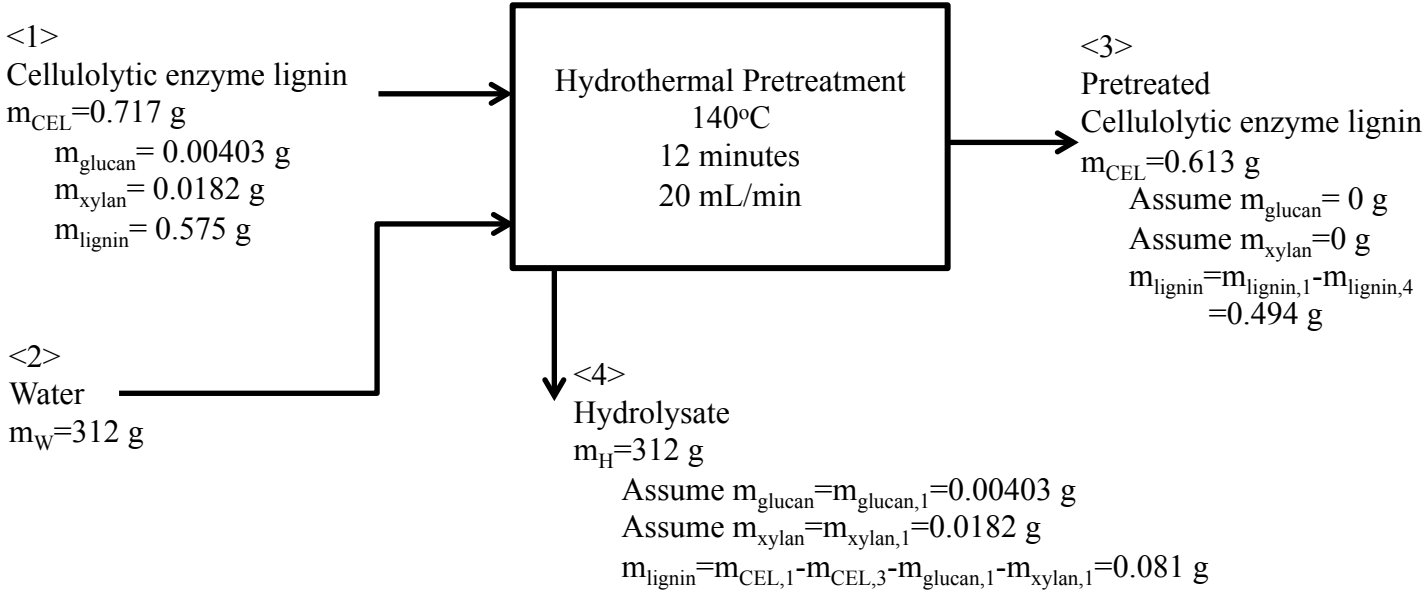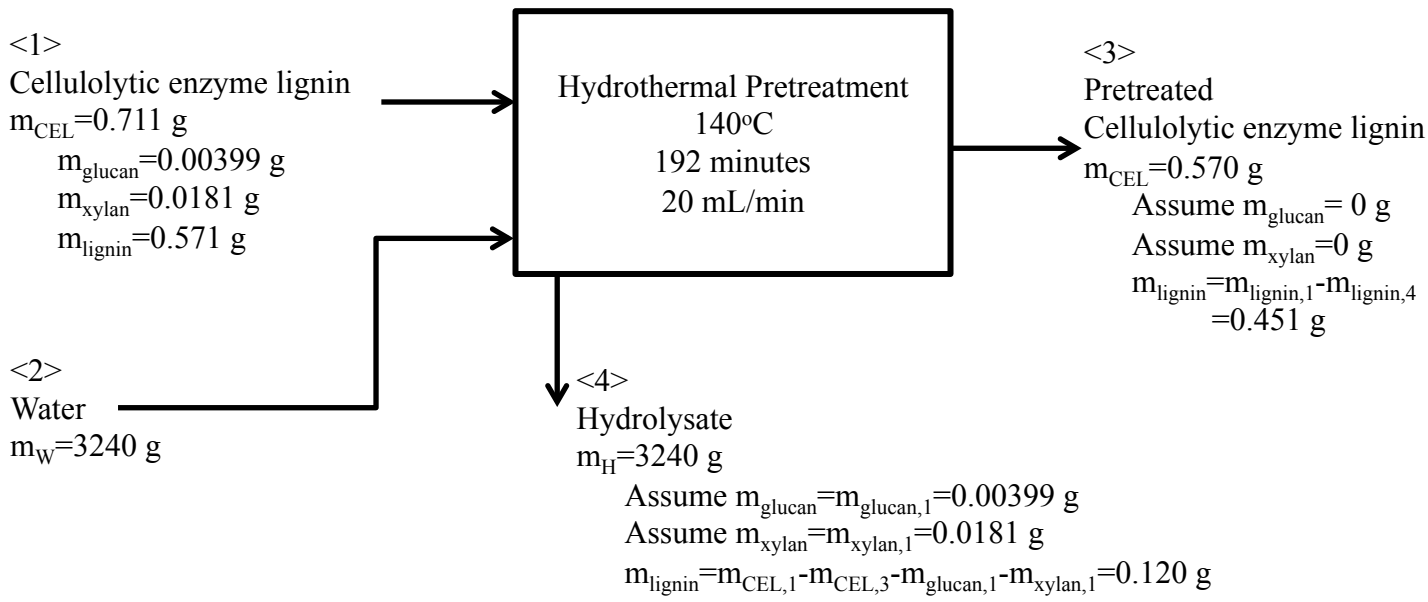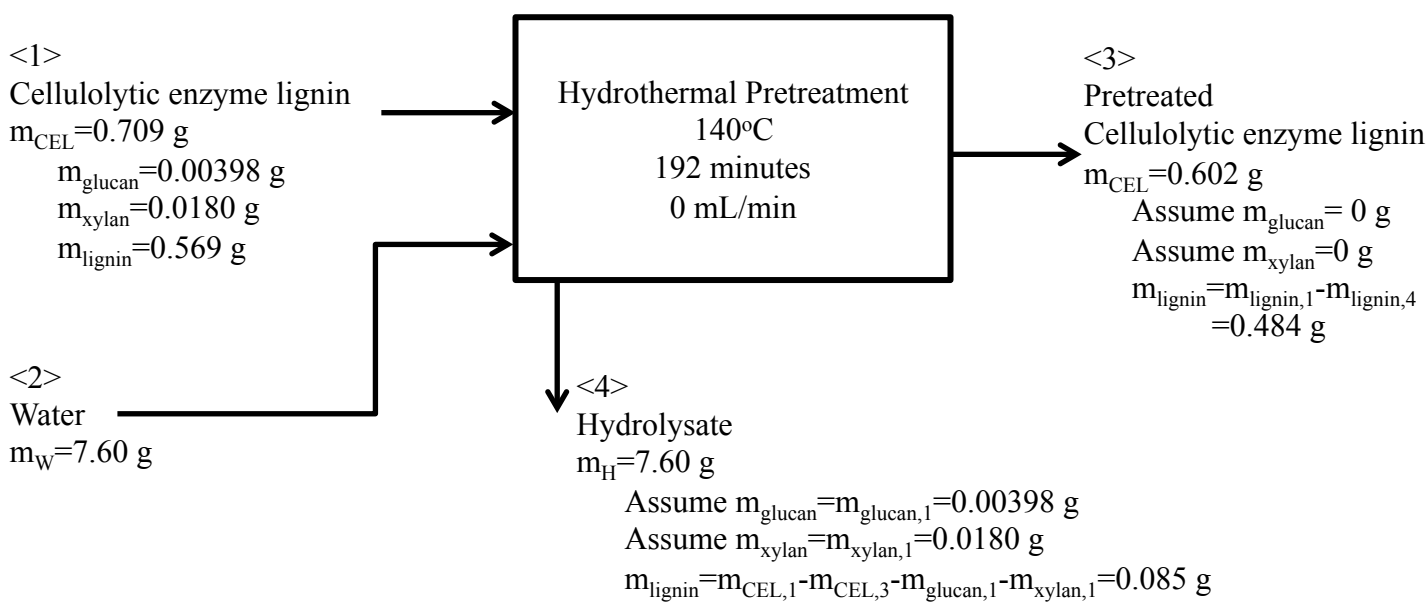

Supplement: Additional file 1 — Mass balances for the pretreatment of cellulolytic enzyme lignin at 140°C. Additional file 1 summarizes the glucan, xylan, and Klason lignin mass balances for the pretreatment of cellulolytic enzyme lignin (CEL) at 140°C. The CEL fused during pretreatment to form a single solid particle therefore it was not possible to perform a compositional analysis on the residual solids. The concentrations of sugars in the hydrolysate were too low to accurately measure. However, as no carbohydrate signals were present in the HSQC-NMR spectra of the pretreated CEL (Figure 4) and no lignin was detected in holocellulose, the cellulose-hemicellulose fraction of the P. trichocarpa x P. deltoides samples, pretreated at the same conditions, it was assumed that the glucan and xylan were completely removed during pretreatment. Therefore the initial mass of glucan and xylan was substracted from the change in solid mass to determine the mass of lignin removed. [file 1754-6834-6-110-S1.pdf]

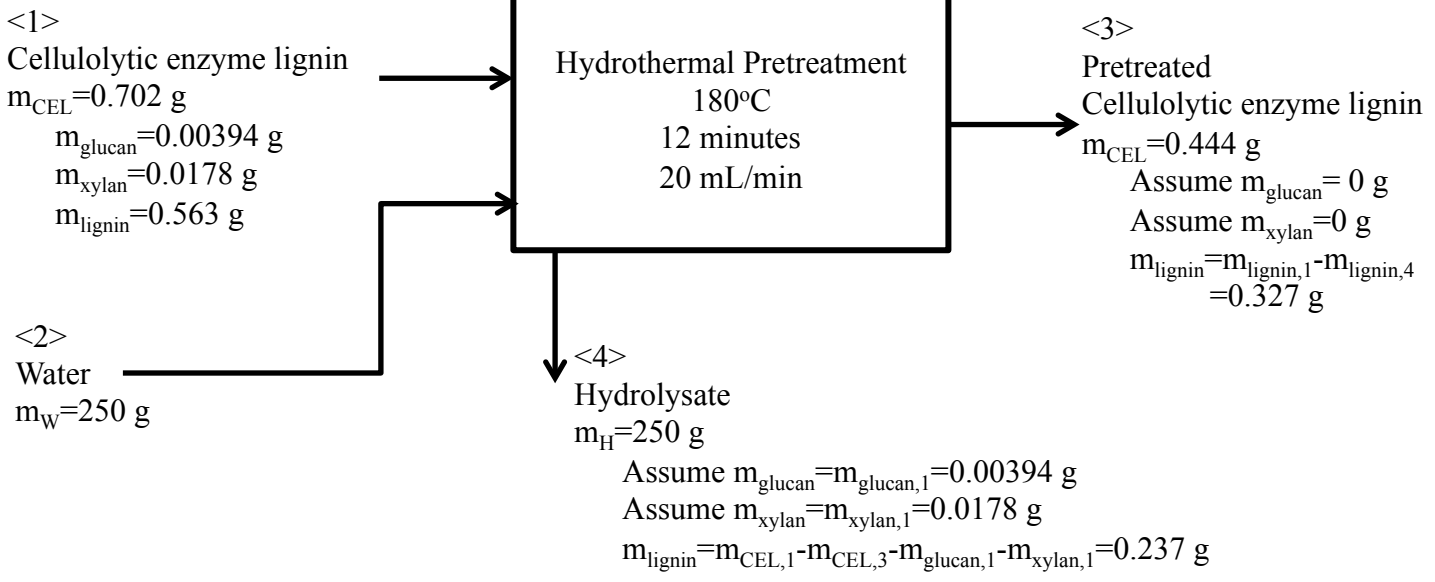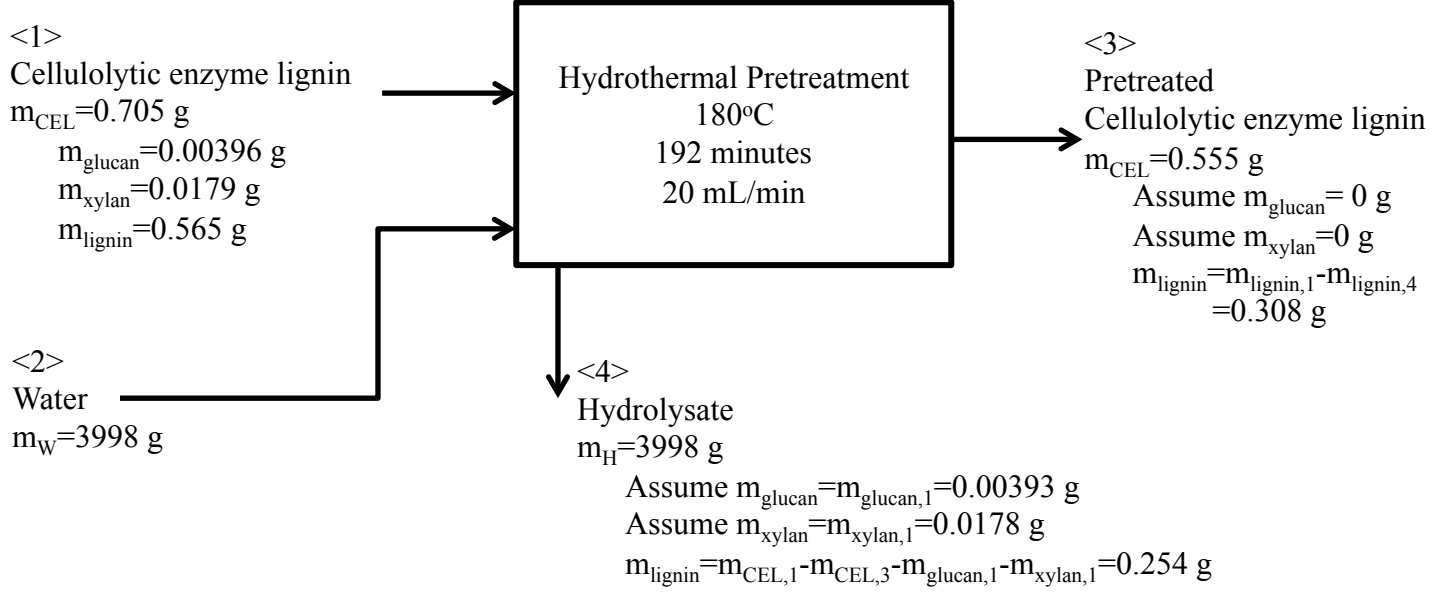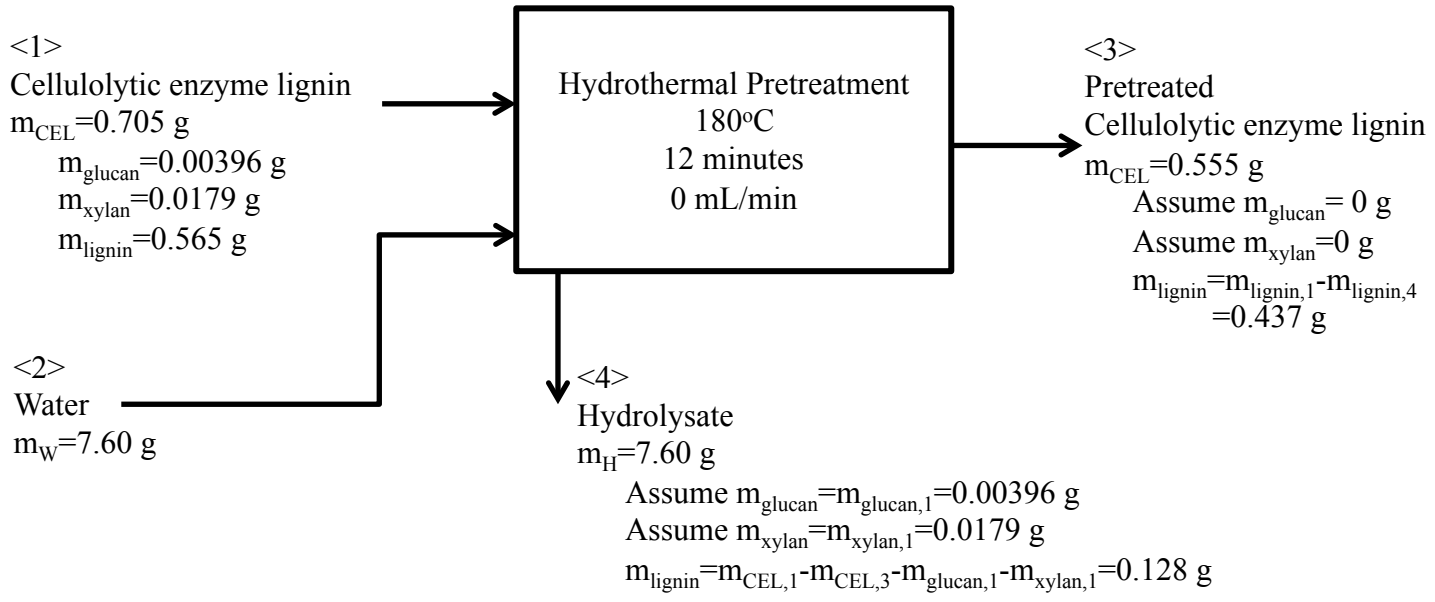

Supplement: Additional file 2 — Mass balances for the pretreatment of cellulolytic enzyme lignin at 180°C. Additional file 2 summarizes the glucan, xylan, and Klason lignin mass balances for the pretreatment of cellulolytic enzyme lignin (CEL) at 180°C. The mass balances were calculated using the same process described for Additional file 1. [file 1754-6834-6-110-S2.pdf]

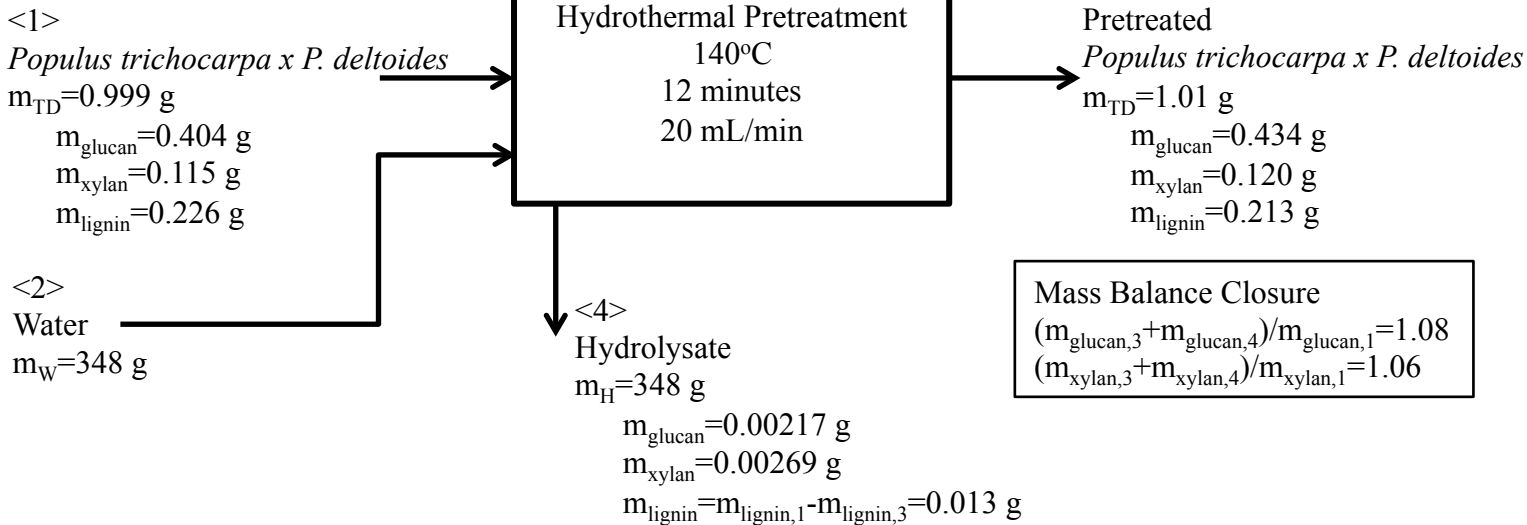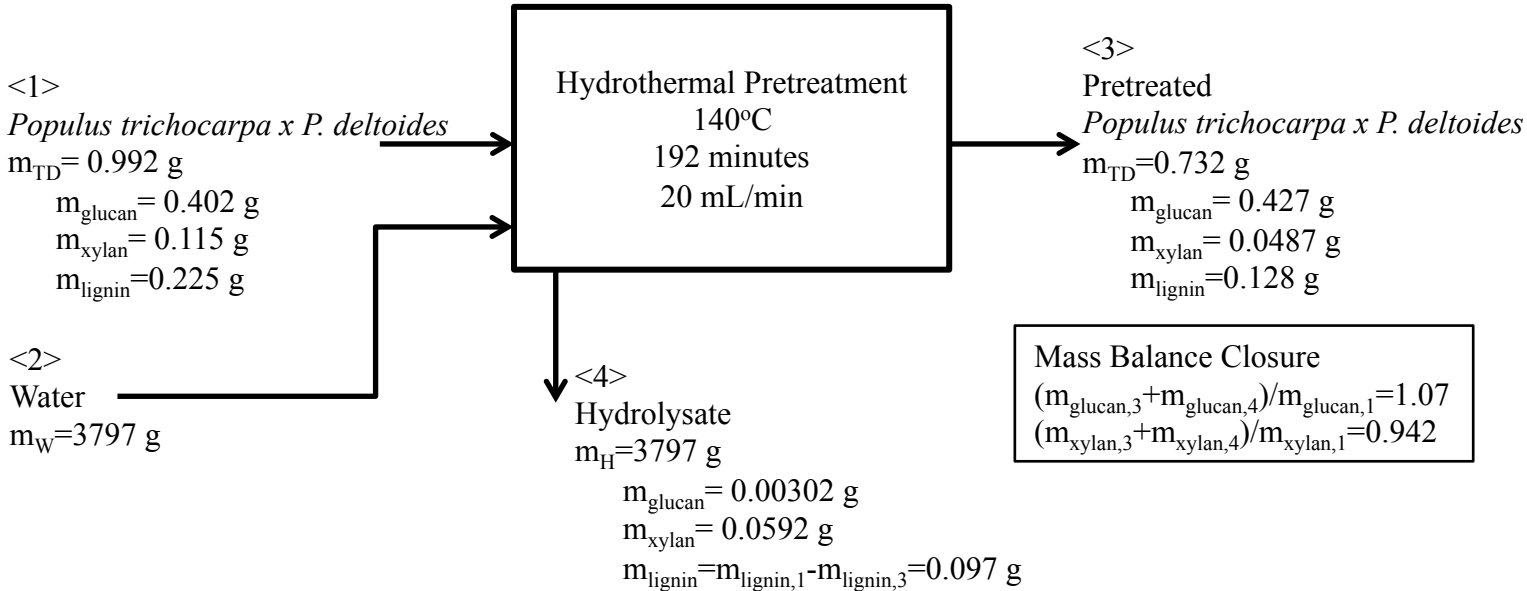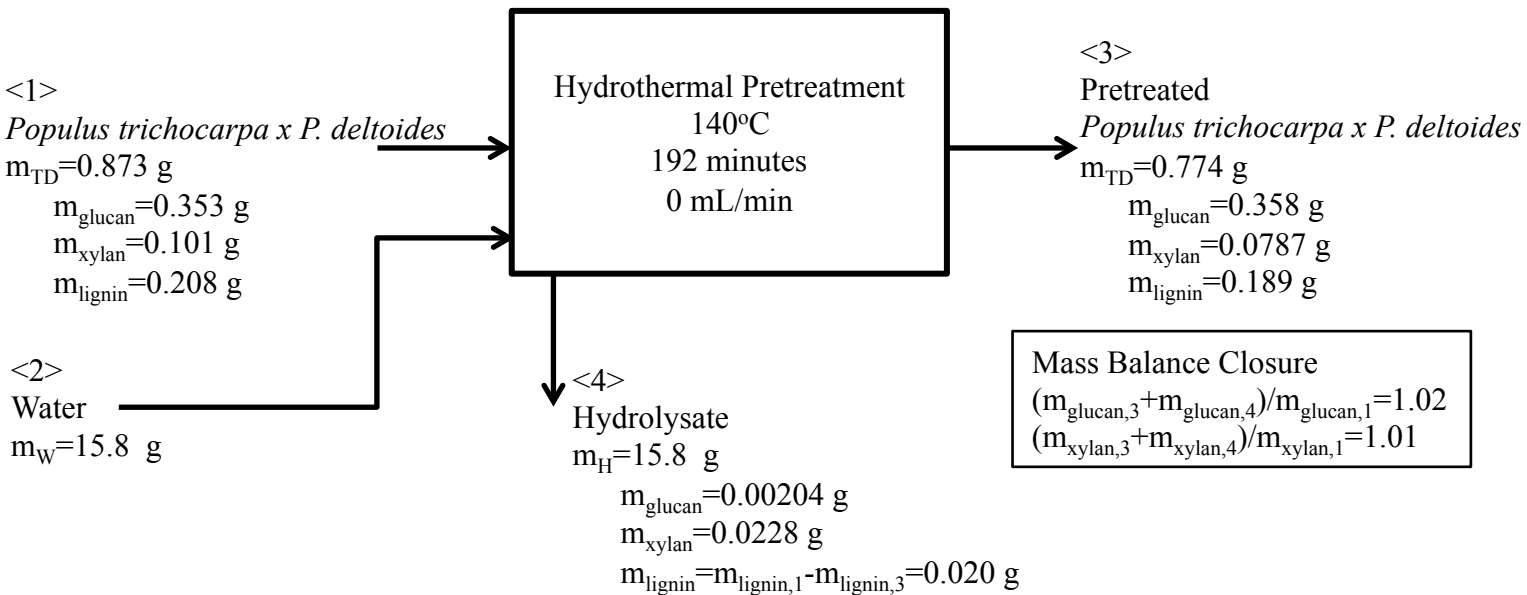

Supplement: Additional file 3 — Mass balances for the pretreatment of Populus trichocarpa x P. deltoides at 140°C. Additional file 3 summarizes the glucan, xylan, and Klason lignin mass balances for the pretreatment of Populus trichocarpa x P. deltoides at 140°C. The mass of lignin removed was calculated as the difference between the Klason lignin in the untreated and pretreated solids. [file 1754-6834-6-110-S3.pdf]

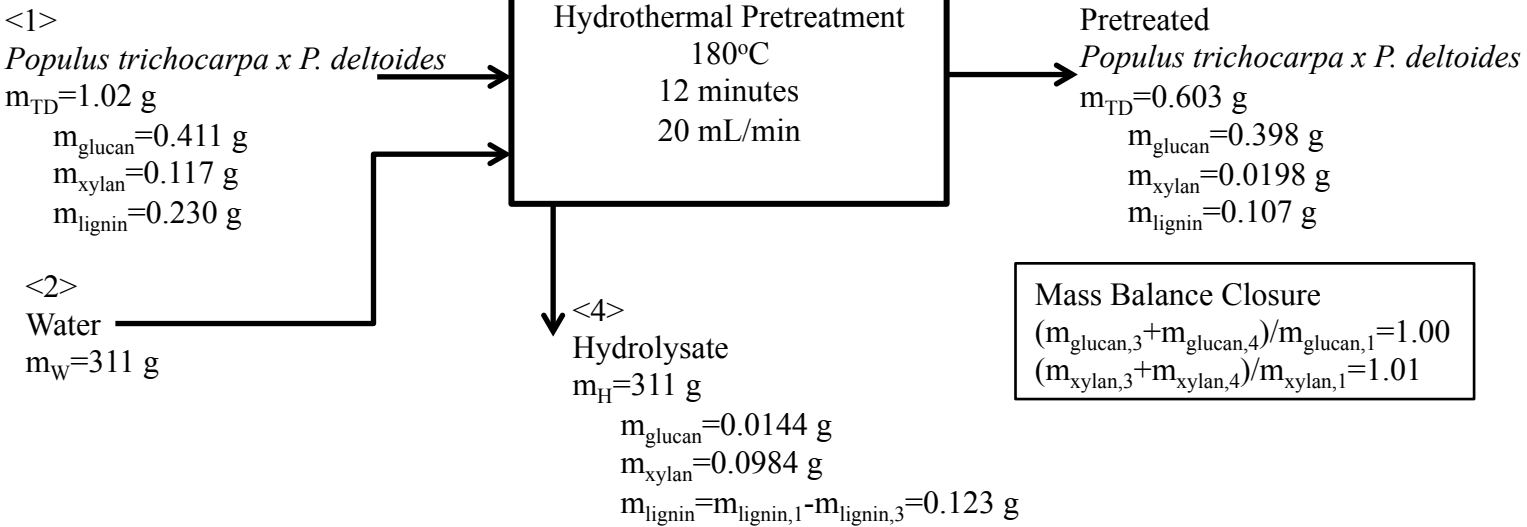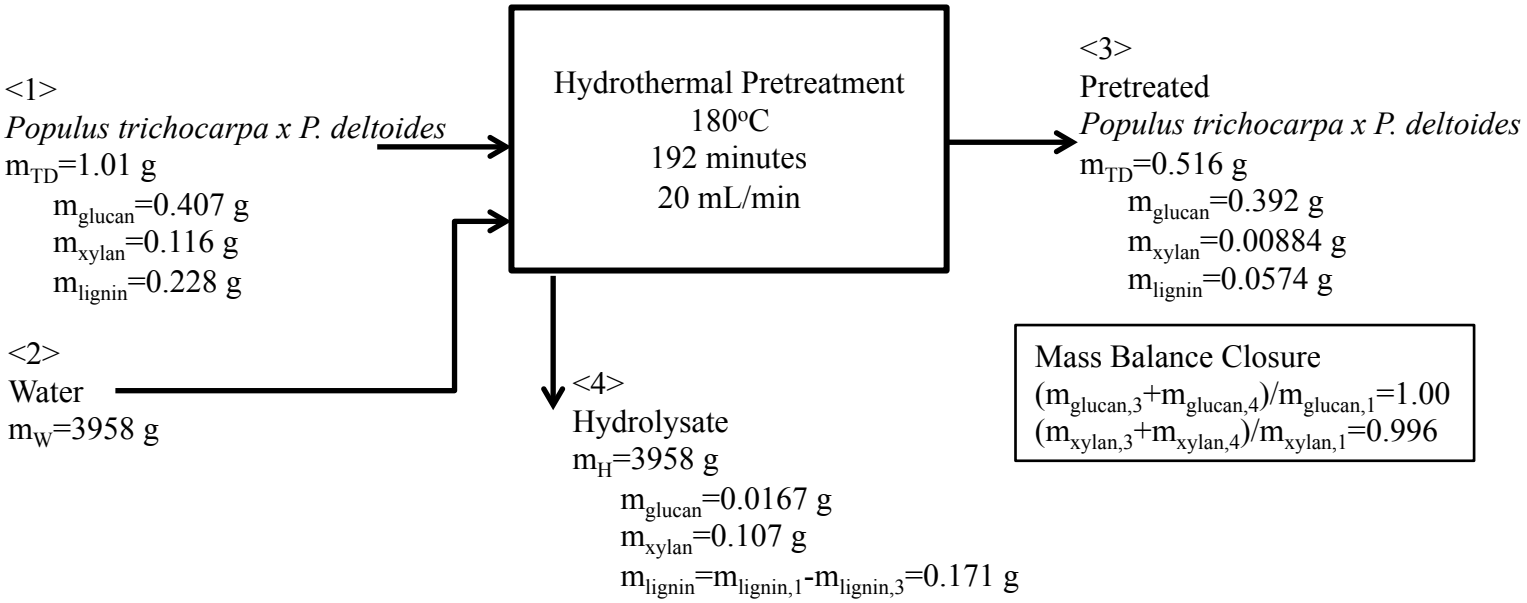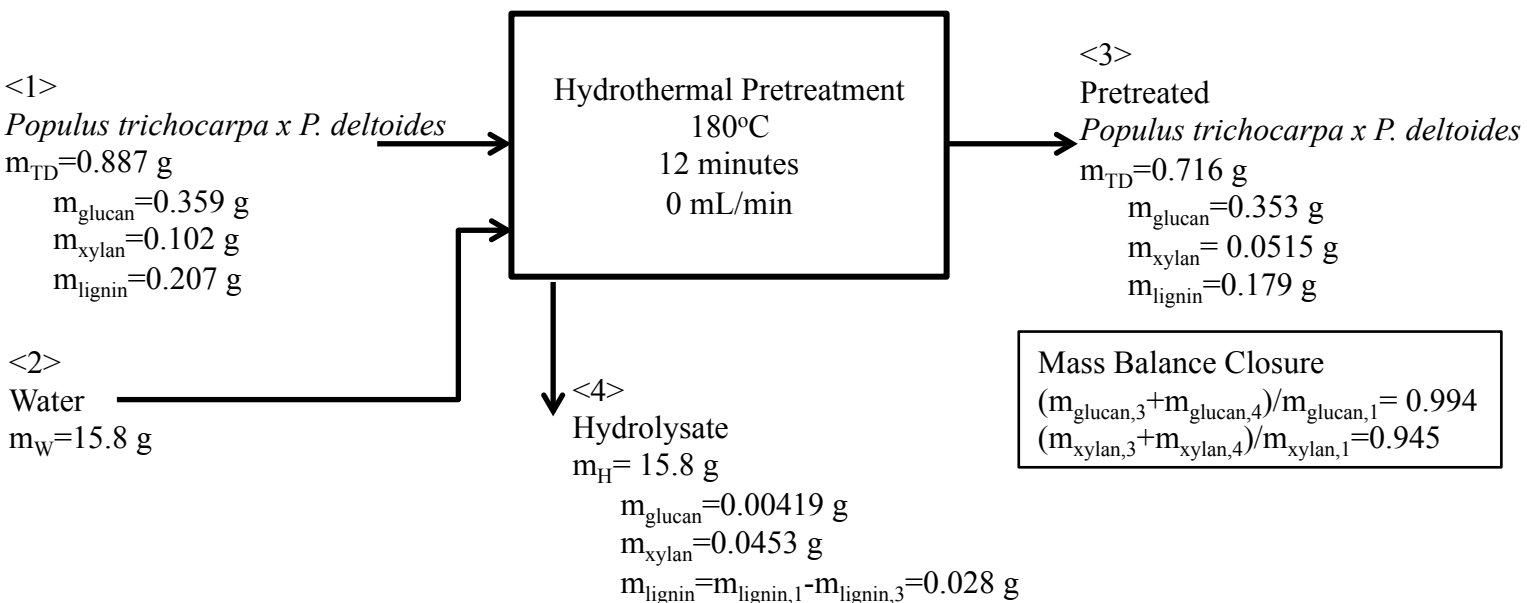

Supplement: Additional file 4 — Mass balances for the pretreatment of Populus trichocarpa x P. deltoides at 180°C. Additional file 4 summarizes the glucan, xylan, and Klason lignin mass balances for the pretreatment of Populus trichocarpa x P. deltoides at 180°C. The mass of lignin removed was calculated as the difference between the Klason lignin in the untreated and pretreated solids. [file 1754-6834-6-110-S4.pdf]
